# Horseshoe adrenal gland associated with type 1 diastematomyelia in an asymptomatic adult

**DOI:** 10.1259/bjrcr.20200188

**Published:** 2021-01-28

**Authors:** Ruhaid Khurram, Faisal Ahmadi, Raunak Poonawala, Ahmad Samim Yasin

**Affiliations:** 1 Department of Radiology, Royal Free Hospital, Royal Free London NHS Foundation Trust, London, UK; 2 University of Sheffield Medical School, University of Sheffield, Sheffield, UK

## Abstract

A horseshoe adrenal gland is a rare congenital anomaly found almost exclusively in neonates and infants based on autopsy studies. It is a term used to describe a solitary adrenal gland situated in the midline, posterior to the inferior vena cava and abdominal aorta. To date, in the literature, there have been very few cases documented in adults and they have also been reported to be associated with other co-existing intra-abdominal, vascular and vertebral congenital anomalies. We describe a rare case of an asymptomatic adult patient who was incidentally found to have a horseshoe adrenal gland as well as a Type 1 diastematomyelia.

## Introduction

A horseshoe adrenal gland is an extremely rare congenital anomaly characterised by the presence of a solitary fused adrenal gland. It is a finding documented almost exclusively in fetal, neonatal and infant autopsy studies and to date, there have only been a total of six prior cases reported in adults.^1–6^ It is often found incidentally on imaging, *e.g.* ultrasound, CT or MRI in otherwise asymptomatic adults. In several cases, patients with a horseshoe adrenal gland have other incidental developmental abnormalities such as: asplenia, neural tube defects, renal, vertebral and diaphragmatic anomalies.^3,6^

Diastematomyelia, also referred to as a split cord malformation, describes a congenital split within the spinal cord and is a type of spinal dysraphism. This accounts for approximately 5% of all spinal congenital defects and is classified into two types. Type 1 is often symptomatic and occurs in the presence of a midline osseous or fibrous spur and a duplicated dural sac. On the contrary, Type 2 has a single dural sac containing two hemicords and is often asymptomatic and identified incidentally.^7^

We report a case of a 38-year-old asymptomatic male who was incidentally found to have a horseshoe adrenal gland and a concomitant Type 1 diastematomyelia on a routine CT abdomen and pelvis scan for surgical planning.

### Case description

A 38-year-old male underwent an emergency laparotomy with Hartmann’s procedure and peritoneal lavage following an admission to hospital in March 2019 for a perforated sigmoid colon secondary to rectal packing. Following the complicated surgery, he was left with an end colostomy and a large abdominal wall defect due to a failure of closure of the anterior abdominal wall. His past medical history includes HIV for which he takes dual-antiretroviral therapy: darunavir 800 mg once daily and ritonavir 100 mg once daily. The patient had a difficult post-operative recovery complicated by hospital acquired pneumonia and intra-abdominal fluid collections both of which were managed conservatively with antibiotics.

**Figure 1.**
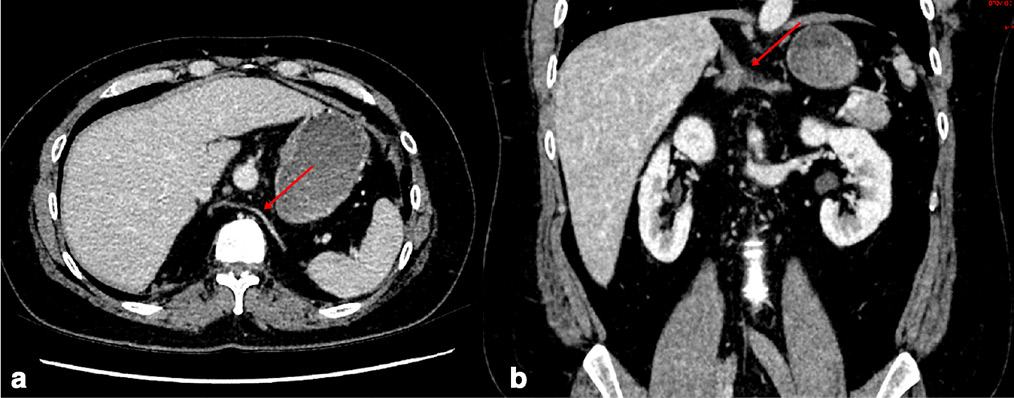
**(**a) Axial and (b) Coronal reformats of a CT abdomen and pelvis scan with contrast in the portal venous phase demonstrating the unique horseshoe-shaped fused adrenal glands (red arrows).

**Figure 2.**
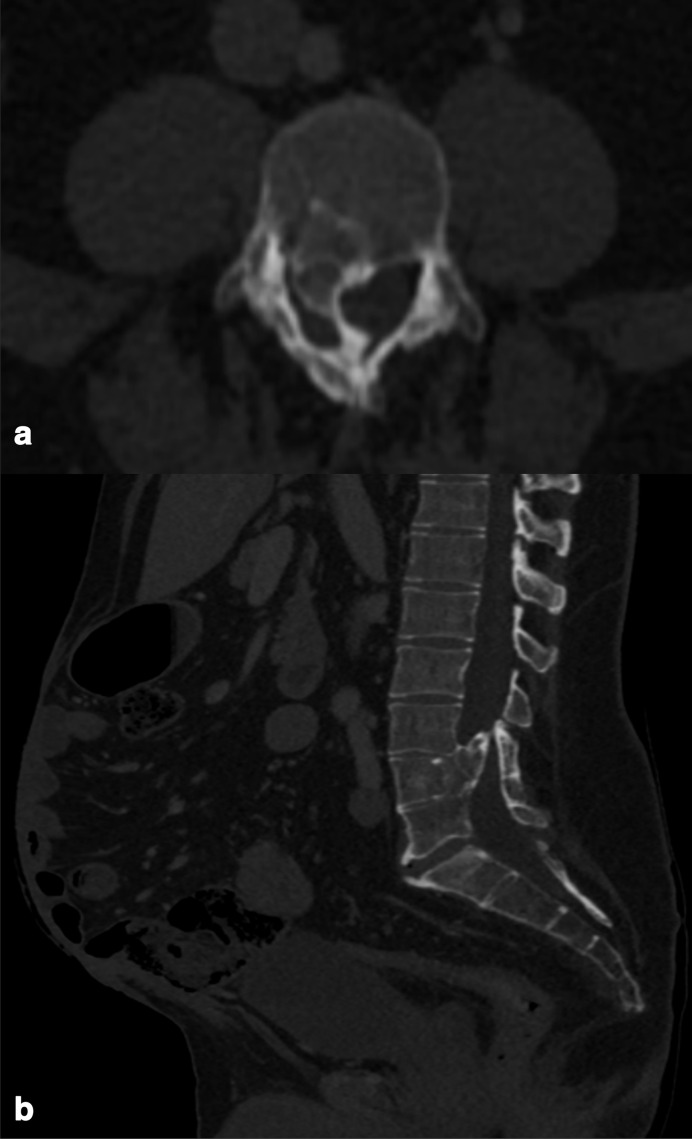
**(**a) Axial and (b) sagittal reformats on bone windows (level 800 HU, width 2000 HU) depicting a developmental anomaly of the L4 vertebra in which the vertebral body and spinous process are fused together by a bony spur. This is consistent with a Type 1 diastematomyelia. Note is made of the large iatrogenic postoperative anterior abdominal wall defect awaiting surgical reconstruction which is best appreciated on the sagittal view.

The patient is now well and is currently awaiting reversal of his Hartmann’s procedure and an abdominal wall reconstruction surgery. On a recent interval CT abdomen and pelvis scan performed for pre-operative planning, he was noted to have a rare congenital anatomical anomaly – a solitary horseshoe adrenal gland with the bridge situated posterior to the inferior vena cava and aorta as well as a retroaortic left renal vein (Figure 1). Interestingly, there was also a developmental anomaly of the L4 vertebra in which the vertebral body and spinous process were fused together by a bony spur. This was consistent with a Type 1 diastematomyelia (Figure 2). The remainder of the solid abdominal organs were apparently unremarkable. The patient did not exhibit any clinical or biochemical features relating to adrenal insufficiency or hyperactivity, nor did he demonstrate features of myelopathy or cauda equina syndrome.

## Discussion

A horseshoe adrenal gland or a butterfly adrenal gland is a solitary adrenal gland that is situated in the midline, posterior to the inferior vena cava and the abdominal aorta. It is almost exclusively found in infants or neonates, however, a few cases have been reported in adults. It is postulated that its existence is due to a failure of midline structures to separate while another theory states that it is due to a failure of laterality because of a disturbed pattern of formation during blastogenesis.^1^ This rare congenital anomaly is associated with multiple malformations that involve the cardiovascular, genitourinary and central nervous system. Among them, the most common reported one is asplenia (52%), followed by neural tube defects (37%), renal abnormalities (29%) and diaphragmatic defects (1%).^3^ In literature, there have only been six cases of horseshoe adrenal glands reported in adults, each with variable number of associated congenital anomalies (Table 1).^1–6^

**Table 1.** A summary of previous case reports identifying horseshoe adrenal glands in adults with associated developmental variations

Author	Age	Sex	Imaging modality	Renal tract variant	Spinal variant	Vascular variant	Other variant
Feldmann et al^1^	60	Male	CT	None	None	None	Bilateral absence of posterior and lumbar diaphragmatic muscle fibres with ectopic “floating” crura Bilateral herniation of retroperitoneal fat into posterior thorax
de Visschere et al^2^	22	Female	CT	Neurogenic bladder with hydronephrosis of right kidney Resected left kidney for renal atrophy	1. Congenital lumbosacral spina bifida with meningomyelocoele	None	1. Rudimentary uterus with imperforate hymen
Ditkofsky et al^3^	51	Female	CT MR	None	1. T9 butterfly vertebra	1. Retro-aortic left renal vein	Posterior midline diaphragmatic hernia Underdeveloped paraspinal musculature at level of diaphragmatic defect Discontinuous fusion of lateral aspects of seventh-9th ribs Bifid 10th rib
Maldonado et al^4^	44	Male	CT	Congenital agenesis of right kidney Chronic left ureteropelvic junction stricture Congenital absence of right kidney and ureter	Congenital fusion of L4-L5 facets Limbus vertebra at L4.	None	1. Congenital absence of right seminal vesicle
Hursoy et al^5^	61	Male	CT PETCT MR	1. Unilateral right renal agenesis	None	None	Congenital absence of right seminal vesicle Diaphragmatic defect at level of aortic hiatus
Romano et al^6^	70	Male	CT	None	Scoliosis T9 butterfly vertebra Split lumbosacral thecal sac at L5	Left common carotid artery arising from innominate artery Absent coeliac trunk	None

The adrenal glands play a vital role in hormone regulation and physiological homeostasis. The cortex and medulla of the adrenal gland have independent embryonic ontogenesis.^8^ The adrenal cortex is derived from an aggregate of mesenchymal cells (developed from mesoderm) adjacent to the dorsal mesentery during the fourth week after fertilisation. Its development is mainly regulated by adrenocorticotrophic hormone. These cells eventually envelop the cell mass which forms the adrenal medulla.^9^ The adrenal medulla is derived from neural crest cells, which in turn is derived from ectoderm. These cells migrate from the dorsal midline lateral to the neural tube. Genes implicated in the embryogenesis of the adrenal glands include Sox, PAX and Sonic hedgehog.^3^

Although the embryologic origin of horseshoe adrenal gland is not yet completely understood, it is believed to occur in the week 5 to 7 of embryogenesis.^8^ Two of the cases described resulted associated diaphragmatic anomaly^3,5^ which was hypothesised to be due to a shared embryologic precursor of the adrenal cortex, diaphragm and vertebra: the mesoderm. It was proposed by Strouse et al^10^ that the adrenal gland starts as a primordial mesodermal mass that subsequently separates. Therefore, the formation of a fused horseshoe adrenal gland may be secondary to a failure of separation from this mass into the left and right adrenal glands. However, this theory is challenged by some authors who hypothesise that complex cell signalling involved in cell migration, proliferation and differentiation can be the main culprit in formation.^3^

Asymptomatic Type 1 diastematomyelia is rare and has been incidentally detected on neuroimaging in a few documented cases. It is commonly associated with tethered cord syndrome, accounting to 10–38% of diagnoses. The diagnostic modality of choice is MRI as it is able to accurately delineate the extent of the dural and spinal cord separation.^11,12^

Similar to our case study, vertebral ones are common concomitant abnormalities found in patients with a horseshoe adrenal gland.^2–4,6^ These can be explained by the persistent notochord hypothesis, which is based on midline mesodermal adhesions between the ectoderm and endoderm which cause the split and re-fusion of the notochord around the adhesion. This theory could therefore explain the concurrent diaphragmatic defects, fused vertebral transverse processes and ribs. However, from a developmental point of view, PAX and Sox transcription factors are also crucial for organogenesis and proliferation of the diaphragm, adrenal gland and skeletal system.^13,14^ Absence or mutation of these factors have been linked to abnormalities in the aforementioned organs.^3^ Further research is required to elicit the exact role of these factors in these anatomic pictures.

## Learning points

1. Horseshoe adrenal gland is an extremely rare anatomical variant in adults and has been reported to be associated with vascular, spinal and intra-abdominal developmental anomalies.
2. It is often asymptomatic and detected incidentally on routine imaging.
3. Awareness and appropriate identification of this anomaly and associated variants are essential by radiologists for a correct radiological report.
